# Low Relative Sit‐to‐Stand Power Is Associated With the Development of Adverse Health Outcomes: A 5‐Year Longitudinal Study

**DOI:** 10.1002/jcsm.13852

**Published:** 2025-06-16

**Authors:** Mikel Garcia‐Aguirre, Ivan Baltasar‐Fernandez, Julian Alcazar, Ana Alfaro‐Acha, F. A. Bareiro‐Quiñonez, Ignacio Ara, Leocadio Rodriguez‐Mañas, Francisco J. Garcia‐Garcia, Luis M. Alegre

**Affiliations:** ^1^ GENUD Toledo Research Group, Faculty of Sport Sciences University of Castilla‐La Mancha Toledo Spain; ^2^ Centro de Investigación Biomédica en red Fragilidad y Envejecimiento Saludable (CIBERFES), Instituto de Salud Carlos III Madrid Spain; ^3^ Grupo Mixto de Fragilidad y Envejecimiento Exitoso UCLM‐SESCAM Universidad de Castilla‐La Mancha‐Servicio de Salud de Castilla‐La Mancha, IDISCAM Toledo Spain; ^4^ Faculty of Health Sciences University of Castilla‐La Mancha Talavera de la Reina Spain; ^5^ Geriatrics Department Hospital Universitario de Toledo Toledo Spain; ^6^ Geriatrics Department Getafe University Hospital Getafe Spain

**Keywords:** chair stand, cognitive function, disability, frailty, successful aging

## Abstract

**Background:**

Relative sit‐to‐stand (STS) power has emerged as a key biomarker of aging due to its strong association with adverse health outcomes such as frailty or disability. Thus, this study aimed to evaluate the association between low baseline relative STS power with the development of adverse health outcomes.

**Methods:**

A total of 839 community‐dwelling older adults (65–91 years; 42% men) from the Toledo Study for Healthy Aging were assessed at baseline and after 5 years of follow‐up. Relative STS power was assessed using the 30‐s STS test and Alcazar's equation. Adverse conditions considered encompassed frailty (evaluated using the frailty trait scale 5 [FTS5] or frailty phenotype [FP]), disability in basic (BADL; Barthel index) and instrumental activities of daily living (IADL; Lawton and Brody scale), cognitive impairment (mini‐mental state examination), depression (geriatric depression scale) and medication use.

**Results:**

At baseline, people with low relative STS power (461 participants) had significantly higher FTS5 (+5.9 points), FP (+0.56 criteria), disability in BADL (−0.1 points) and IADL (−0.7 points), cognitive impairment (−1.3 points) and medication use (+0.9 medications) than older adults with normal relative STS power (all *p* < 0.05). In contrast, no significant differences were observed at baseline in GDS (*p* > 0.05). Low baseline relative STS power was significantly associated with the incidence of frailty FTS5 (OR [95% CI] = 2.51 [1.26–5.03]; *p* = 0.009), disability in BADL (OR [95% CI] = 1.70 [1.13–2.56]; *p* = 0.011) and IADL (OR [95% CI] = 1.79 [1.06–3.02]; *p* = 0.030) and increased medication use (OR [95% CI] = 1.51 [1.10–2.07]; *p* = 0.011) during the follow‐up. No association was found with the incidence of frailty by FP (OR [95% CI] = 1.71 [0.75–3.93]; *p* = 0.202), depression (OR [95% CI] = 1.29 [0.85–1.98]; *p* = 0.236) or cognitive impairment (OR [95% CI] = 1.38 [0.86–2.21]; *p* = 0.178).

**Conclusion:**

Participants with low relative STS power exhibited worse baseline and 5‐year follow‐up values in frailty, BADL and IADL disability, cognitive impairment and medication intake. Low relative STS power was also associated with a higher probability of future frailty, disability in BADL and IADL and increased medication use.

## 1 Introduction

As life expectancy increases globally [1], healthcare systems are faced with the challenge of managing a growing older population [2], with age‐related conditions becoming more prevalent [3]. In this context, understanding and predicting the progression of age‐related physiological changes has become a priority in healthcare. The ability to identify individuals at risk of accelerated aging or age‐related diseases enables the development of targeted interventions that can improve health outcomes in older populations. Early detection and intervention strategies may have the potential to reduce healthcare costs and minimize the impact of aging. In recent years, the identification of biomarkers has become essential for predicting aging trajectories, focusing on physiological and genetic markers such as hormonal levels or telomere length linked to age‐related disease [4]. However, these biomarkers are often expensive and challenging to measure, which calls for the search of more feasible and functional markers [5]. The aging process negatively affects the musculoskeletal system [6], leading to a deterioration of muscle function and structure, finally worsening the ability to perform motor tasks [7]. One of the main indicators of muscle function is muscle power, defined as the product of force and velocity or the rate at which mechanical work is done. Muscle power has been recognized as a critical biomarker, given its more rapid and pronounced age‐related decline and stronger association with functional performance compared to other neuromuscular variables [8, 9]. These findings highlight the increasing relevance of assessing muscle power in clinical settings. However, until recently, this assessment was expensive and relatively complex to implement in clinical and other health‐related settings. The introduction of the sit‐to‐stand (STS) muscle power test [10] implies a significant advance in this sense, since it represents an inexpensive, valid, reliable and feasible test to assess muscle power in the clinical setting [10]. Importantly, cross‐sectional studies have shown that lower levels of STS power normalized by body mass (i.e., relative STS power) have been associated with increased odds of frailty, disability and other adverse health outcomes [11, 12, 13]. While these studies provide valuable insights, none have conducted longitudinal assessments to establish the clinical significance of the STS muscle power test in terms of its association with future negative outcomes related to aging. There is a critical need for longitudinal research to validate the prognostic value of STS power over time, as this would provide stronger support for its status as a key biomarker of aging. To our knowledge, this is the first study examining the long‐term effects of low relative STS power on various adverse health outcomes over a 5‐year follow‐up period. Therefore, the main aim of this study was to evaluate the association between low baseline relative STS power with the development of frailty, disability, cognitive impairment, depression and increased medication use.

## 2 Materials and Methods

### 2.1 Study Design and Participants

**FIGURE 1:**
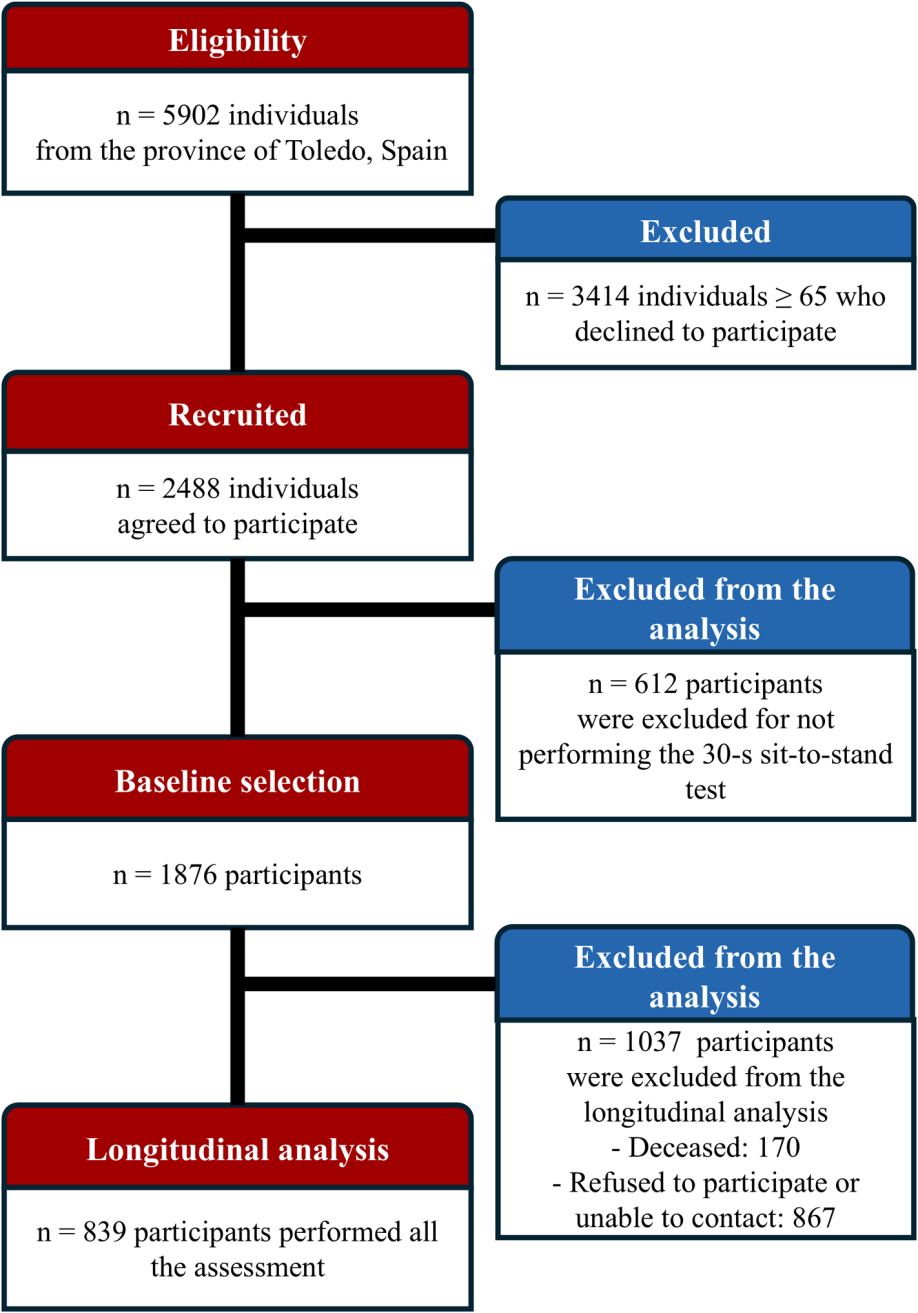
Participant flowchart.

This study is a prospective cohort study conducted within the framework of the first (2006–2009) and second (2011–2013) waves of the Toledo Study of Healthy Aging (TSHA). Data were obtained from randomly selected community‐dwelling older adults aged 65 and above from the municipal census of Toledo through a two‐stage random sampling process. The detailed methodology of the TSHA has been previously reported [14]. A total of 1876 participants were evaluated at baseline. During the follow‐up period, 1037 participants were lost, primarily due to mortality (*n* = 170) and because they declined to participate or were unable to contact them (*n* = 867) (Figure 1). Finally, 839 participants (356 men and 483 women) were included in this study (Table 1). To mitigate potential bias, baseline characteristics of participants who completed the follow‐up were compared with those who were lost, as detailed in Table S1. The Clinical Research Ethics Committee of the Toledo Hospital (Spain) approved both waves of the study protocol (approval dates: 30/03/05, reference number 22; and 15/07/10, reference number 93). All participants signed an informed consent, and the study was performed according to the ethical standards of the 1964 Declaration of Helsinki and its subsequent amendments.

**TABLE 1:** Baseline characteristics of study participants.

	All participants (*N* = 839)	Men (*n* = 356)	Women (*n* = 483)
	Mean ± SD	Mean ± SD	Mean ± SD
Age (years)	74.2 ± 5.4	73.8 ± 5.1	74.5 ± 5.5
Follow‐up time (years)	5.1 1.1	5.1 1.1	5.2 1.2
Weight (kg)	73.0 ± 12.6	77.1 ± 11.8	70.0 ± 12.2*
Height (m)	1.58 ± 0.08	1.64 ± 0.07	1.53 ± 0.06*
BMI (kg.m^−2^)	29.3 ± 4.7	28.4 ± 3.9	29.9 ± 3.9
Relative STS power (W·kg ^−1^ )	2.19 ± 0.83	2.76 ± 0.88	2.08 ± 0.71*
Low relative STS power, *n* (%)	461 (54.9%)	179 (50.4%)	282 (58.4%)*
FTS5 (points)	18.9 ± 6.8	16.8 ± 6.2	21.40 ± 7.15*
Frailty FTS5, *n* (%)	161 (21.4%)	32 (10.0%)	129 (30.0%)*
FP (*n* of criteria)	0.71 ± 0.95	0.67 ± 0.93	0.75 ± 0.98
Frailty FP, *n* (%)	46 (6.0%)	14 (4.2%)	32 (7.3%)
Katz index (points)	5.85 ± 0.66	5.93 ± 0.47	5.79 ± 0.76*
Disability in BADL, *n* (%)	143 (17.2%)	37 (10.5%)	106 (22.2%)*
L&B scale (points)	6.38 ± 2.0	5.65 ± 1.88	6.90 ± 1.84*
Disability in IADL, *n* (%)	553 (55.1%)	244 (73.3%)	199 (42.0%)*
GDS (points)	2.34 ± 2.52	1.73 ± 1.85	2.78 ± 2.83*
Depression, *n* (%)	119 (16.1%)	30 (9.7%)	89 (21.2%)*
MMSE (points)	24.2 ± 5.01	25.0 ± 4.4	23.67 ± 5.36*
Cognitive impairment, *n* (%)	129 (17.7%)	41 (13.4%)	88 (20.9%)*
Charlson index (points)	1.73 ± 1.89	1.52 ± 1.8	1.88 ± 1.97
Medications (*n*)	4.7 ± 2.6	4.1 ± 2.5	5.1 ± 2.6

*Note:* *denotes significant differences compared to men (*p* < 0.05). The number of participants evaluated for some variables were as follows: 752 (322 men and 430 women) for the FTS5, 775 (335 men and 440 women) for the FP, 831 (354 men and 477 women) for BADL, 808 (334 men and 474 women) for IADL, 808 (311 men and 420 women) for the GDS and 728 (307 men and 421 women) for the MMSE.

Abbreviations: BADL, basic activities of daily living; FTS5, frailty trait scale short form; FP, frailty phenotype; GDS, geriatric depression scale; IADL, instrumental activities of daily living; L&B scale, Lawton and Brody scale; MMSE, mini‐mental stated examination; STS, sit‐to‐stand.

### 2.2 Anthropometry

Body mass was measured using a scale with a precision of 0.1 kg (Seca 711, Hamburg, Germany), and height was measured using a portable stadiometer with a precision of 1 mm (Medizintechnikseit 1890; KaWe, Asperg, Germany). Participants were measured while wearing light clothes (underwear) and without shoes. Body mass index (BMI) was calculated by dividing body mass by height squared (kg·m^−2^).

### 2.3 Relative STS Power

STS power was assessed using the 30‐s STS test [15]. Participants were instructed to complete the maximum number of STS repetitions within 30 s after the cue “ready, set, go!” on a 0.43‐m standardized chair without armrests. Participants performed the test with their arms crossed over their chest, and the STS repetitions were considered valid when the participant achieved a full standing position and at least touched the chair with their buttocks when sitting. The maximum number of repetitions completed in the 30‐s STS test was recorded, and the Alcazar equation was used to compute absolute STS muscle power [15]:

$$\begin{array}{c} \text{Absolute}\;\mathrm{STS}\;\text{power}\;\left(W\right)=\\ \left(\text{Body mass}\cdot 0.9\cdot g\right)\cdot \frac{\left(\left[\text{Body height}\cdot 0.5\right]-\text{Chair height}\right)}{\left(\frac{30\;s}{\text{number of}\;\mathrm{STS}\;\text{repetitions}}\right)\cdot 0.5} \end{array}$$

Then, relative STS power was calculated by dividing absolute STS power by body mass (W·kg^−1^). Low relative STS power was considered in participants, with a relative STS power of ≤ 2.53 W·kg^−1^ in men and ≤ 2.01 W·kg^−1^ in women, respectively [16].

### 2.4 Adverse Health Outcomes

#### 2.4.1 Frailty

Frailty was evaluated using two different scales: (1) the frailty trait scale short form (FTS5) [17] and (2) the frailty phenotype (FP) [18]. The FTS5 assesses physical frailty across five different domains (physical activity, handgrip strength, BMI, static balance and habitual gait speed), yielding a final score ranging between 0 (lowest frailty) and 50 (highest frailty). A score > 25 points is indicative of frailty [19]. The FP evaluates frailty through five criteria (self‐reported exhaustion, weakness, unintentional weight loss, slowness and low physical activity levels), classifying the participants as frail (2–5 criteria met), prefrail (1–2 criteria met) or robust (0 criteria met) [18].

#### 2.4.2 Disability in Activities of Daily Living

Disability was assessed in both basic (BADL) and instrumental activities of daily living (IADL) using the Katz index of independence in activities of daily living [20] and the Lawton and Brody scale [21], respectively. The Katz index evaluates functional capacity across six BADLs, with scores ranging from 0 (indicating dependence in all BADLs) to 6 (indicating complete independence). The Lawton and Brody scale assesses disability across eight IADLs, with scores ranging from 0 (indicating dependence in all IADLs) to 8 (indicating complete independence). Disability in BADLs and IADLs was defined as a Katz index score of < 6 points and a Lawton and Brody scale score of < 8 points, respectively [20, 21].

#### 2.4.3 Cognitive Impairment, Depression

Cognitive impairment was evaluated using the mini‐mental state examination (MMSE) [22]. This questionnaire comprises 11 items that evaluate six domains of cognitive function (orientation, repetition, verbal recall, attention and calculation, language and visual construction) and result in a score ranging from 0 (lowest) to 30 (highest cognitive function) points. Cognitive impairment was defined as having an MMSE score < 20 [22]. Depression was evaluated through the geriatric depression scale (GDS) [23], a binary‐response questionnaire of 15 items. A score > 5 points on the GDS was indicative of depression [23].

#### 2.4.4 Comorbidities and Medication Usage

Comorbidities were assessed using the Charlson comorbidity index (S3), a validated tool designed to quantify the number and severity of comorbid conditions. Higher scores reflect a greater burden of comorbidities and an associated increased risk of mortality. Additionally, the total number of medications each participant was taking at the time of the study was recorded.

### 2.5 Statistical Analysis

Descriptive data are shown as mean ± standard deviation (SD) for continuous variables and frequencies (*n*, %) for categorical variables. Kolmogorov–Smirnov tests were used to assess the normality of continuous variable distributions, while Levene's tests were applied to evaluate the homogeneity of variances. Student's *t*‐tests for independent samples and *χ* ^2^ tests were used to evaluate baseline differences between men and women in continuous and categorical variables, respectively. Generalized and linear mixed models adjusted for age, sex, comorbidities, educational level and baseline values were utilized to analyse differences in baseline and follow‐up scores of adverse health outcomes between older adults with low relative STS power and those with normal relative STS power. Bonferroni's post hoc tests were used to assess between‐group differences over time. Binary logistic regression analyses adjusted for age, sex, comorbidities and educational level were conducted to evaluate the association of low relative STS power assessed at baseline with the development of adverse health outcomes between baseline and follow‐up, specifically among participants who were free of adverse health outcomes at baseline. All statistical analyses were performed using SPSS v21 (SPSS Inc. Chicago, IL), and the significance level was set at *α* = 0.05.

## 3 Results

The baseline characteristics of the study participants are shown in Table 1. Older women had a higher prevalence of low relative STS power, frailty FTS5, disability in BADL, depression and cognitive impairment than older men (all *p* < 0.05). No significant sex differences were observed in frailty FP, Charlson index or medication intake (all *p* > 0.05). The average follow‐up time of the study was 5.1 ± 1.1 years.

### 3.1 Longitudinal Differences in Adverse Health Outcomes in Individuals With Low vs. Normal Relative STS Power

**FIGURE 2:**
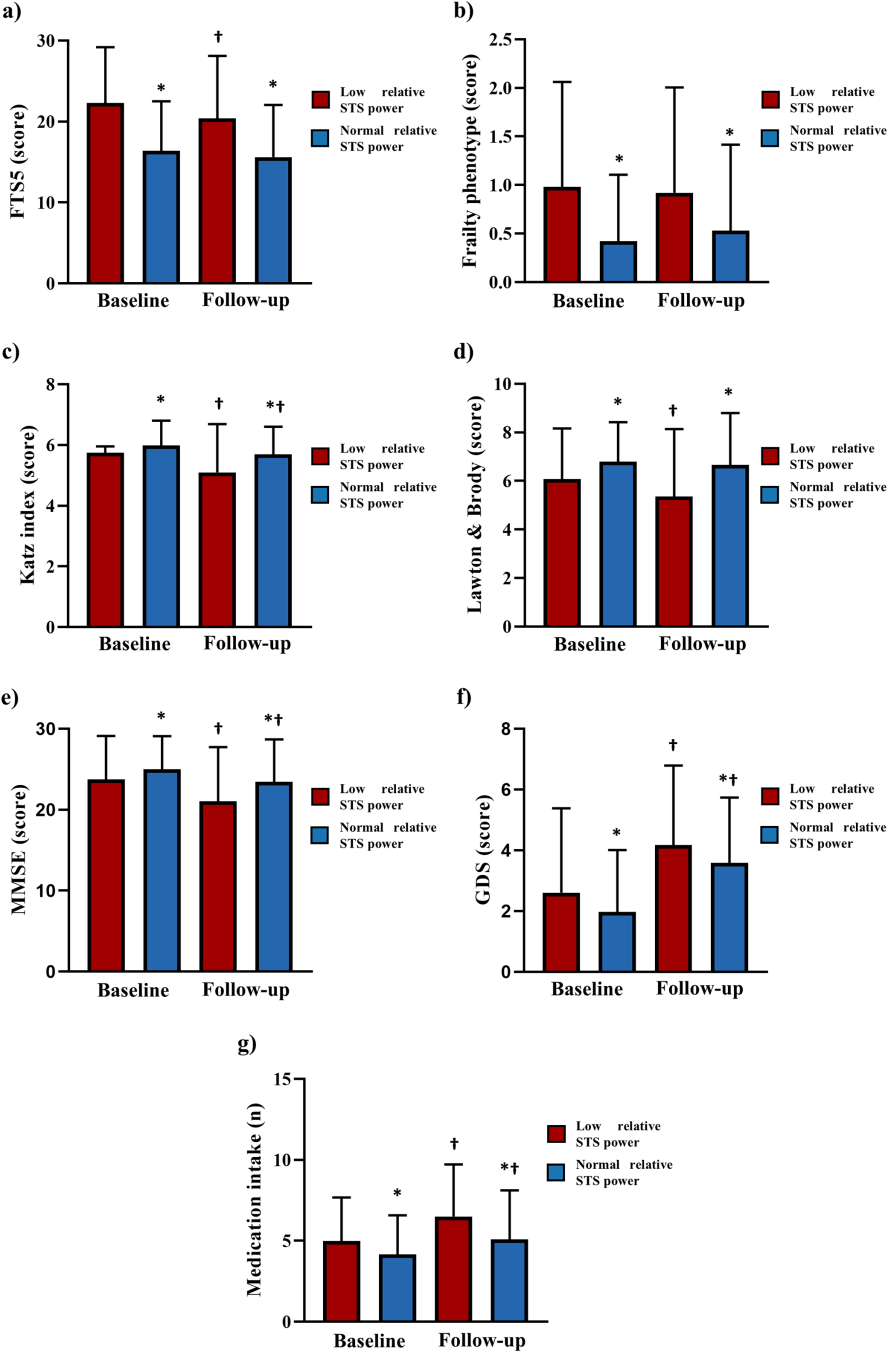
Baseline and longitudinal differences in adverse health outcomes scores between older adults with low relative STS power at baseline and those with normal relative STS power at baseline. *Note:* The analysis was adjusted by age, sex, comorbidities, educational level and baseline values. *Significantly different compared to older adults with low relative STS power within the same time (*p* < 0.05). †Significant differences compared to baseline within the same group (*p* < 0.05). FTS5, frailty trait scale; GDS: geriatric depression scale; MMSE, mini‐mental state examination; STS: sit‐to‐stand.

Figure 2 shows the differences at baseline and after 5 years of follow‐up between the group of older adults with low relative STS power at baseline and the group of older adults with normal relative STS power at baseline. At baseline, older men and women with low relative STS power exhibited higher levels of frailty according to the FTS‐5 (22.3 vs. 16.4 points; *p* < 0.001) and FP (0.98 vs. 0.42 criteria, *p* < 0.001), lower Katz index (5.8 vs. 6.0 points, *p* < 0.01), lower Lawton and Brody scale (6.1 vs. 6.8, *p* = 0.004), lower MMSE (23.7 vs. 25.0 points, *p* = 0.037), higher GDS (2.6 vs. 2.0 points, *p* = 0.001) and higher medication intake (5.0 vs. 4.1 medications, *p* < 0.001), than the group of older adults with normal relative STS power (Figure 2).

After 5 years of follow‐up, the group with low relative STS power at baseline continued to exhibit higher levels of frailty according to the FTS‐5 (20.4 vs. 15.6 points, *p* < 0.001) and FP (0.92 vs. 0.53 criteria, *p* < 0.001), lower Katz index (5.1 vs. 5.7 points, *p* < 0.001), lower Lawton and Brody scale (5.3 vs. 6.7 points, *p* < 0.001), lower MMSE (21.1 vs. 23.4 points, *p* < 0.001), higher GDS (4.2 vs. 3.6 points, *p* = 0.001) and higher medication intake (6.5 vs. 5.1 medications, *p* < 0.001) than the group of older adults with normal relative STS power (Figure 2). The summary of the linear mixed model analysis can be found in Table S2.

In terms of changes, the low relative STS power group showed a reduction in FTS5 (−1.9 points; *p* < 0.001), but no changes were detected in FP (−0.06 criteria; *p* = 0.418). This group also experienced a decline in the Katz index (−0.7 points; *p* < 0.001), the Lawton and Brody scale (−0.7 points; *p* < 0.001) and the MMSE (−2.7 points; *p* < 0.001), alongside an increase in the GDS (+1.6 points; *p* < 0.001) and medication intake (+1.5 medications; *p* < 0.001). In contrast, the normal relative STS power group showed no changes in FTS5 (−0.8 points; *p* = 0.159), FP (+0.11 criteria; *p* = 0.138) or the Lawton and Brody scale (−0.1 points; *p* = 0.433). However, this group exhibited a reduction in the Katz index (−0.3 points; *p* < 0.001) and the MMSE (−1.6 points; *p* < 0.001), as well as an increase in the GDS (+1.6 points; *p* < 0.001) and medication intake (+1.0 medication; *p* < 0.001) (Table S3).

The changes observed between groups were similar regarding FTS5 (*p* = 0.438), FP (*p* = 0.631) and the GDS (*p* = 0.855). Nevertheless, the low relative STS power group experienced a significantly greater reduction in the Katz index (*p* < 0.001), the Lawton and Brody scale (*p* < 0.001) and the MMSE (*p* = 0.024), along with a larger increase in medication intake (*p* = 0.022) compared to the normal relative STS power group (Table S4).

### 3.2 Association Between Baseline Low Relative STS Power and the Development of Adverse Health Outcomes

At baseline, a total of 491 and 572 participants were not frail according to the FTS5 and FP, respectively. In terms of disability, 103 participants had limitations in BADL, and 350 had limitations in IADL. Additionally, 98 participants showed cognitive impairment, and 86 had depression at baseline.

**FIGURE 3:**
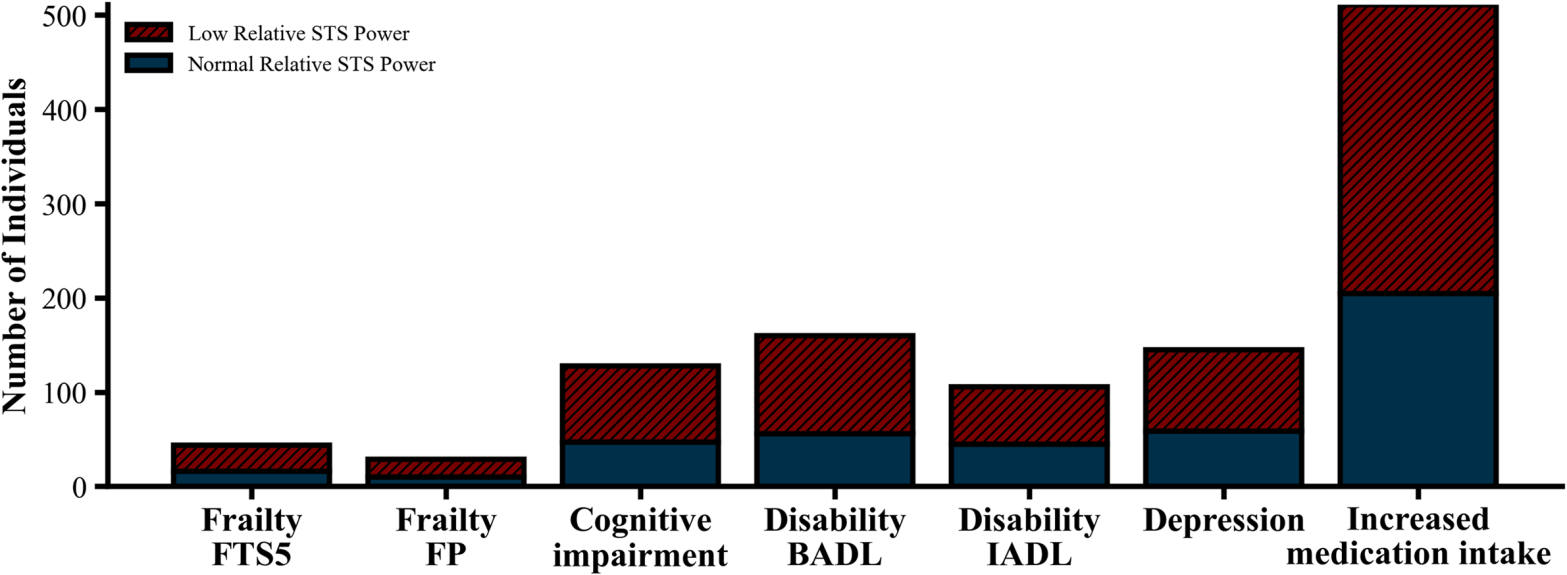
Number of participants without adverse health outcomes at baseline who developed adverse health outcomes after 5 years of follow‐up. *Note:* Striped bars represent participants with low relative STS power at baseline who developed adverse outcomes, while solid grey bars represent those with normal relative STS power at baseline who developed adverse health outcomes. BADL, basic activities of daily living; FP, frailty phenotype; FTS5, frailty trait scale; IADL: instrumental activities of daily living.

After 5 years of follow‐up, 44 participants who were not frail at baseline developed frailty according to the FTS‐5 and 29 according to the FP, of which 28 (63.6%) and 19 (65.5%), respectively, had low relative STS power at baseline (Figure 3). Similarly, 160 participants who had no limitations in BADL at baseline developed BADL disability, and 106 participants who had no limitations in IADL at baseline developed IADL disability, with 104 (65.0%) and 61 (57.5%) of them, respectively, having low baseline relative STS power (Figure 3). Moreover, 128 participants who had no cognitive impairment at baseline developed cognitive impairment and 145 participants who did not have depression at baseline developed depression, with 81 (63.3%) and 56 (38.6%), respectively, having low baseline relative STS power (Figure 3). In addition, after 5 years of follow‐up, 511 participants increased their medication intake, 306 (59.9%) of whom had low relative STS power at baseline (Figure 3).

Unadjusted analysis showed that having low relative STS power at baseline was significantly associated with a higher likelihood of developing frailty according to the FTS‐5 (*p* = 0.015), disability in BADLs (*p* < 0.001), disability in IADLs (*p* < 0.001) and medication use (*p* = 0.016) (Table S5). No significant association was found between low relative STS power at baseline and the development of frailty according to the FP (*p* = 0.077), cognitive impairment MMSE (*p* = 0.095) or depression (*p* = 0.200) (Table S5).

**FIGURE 4:**
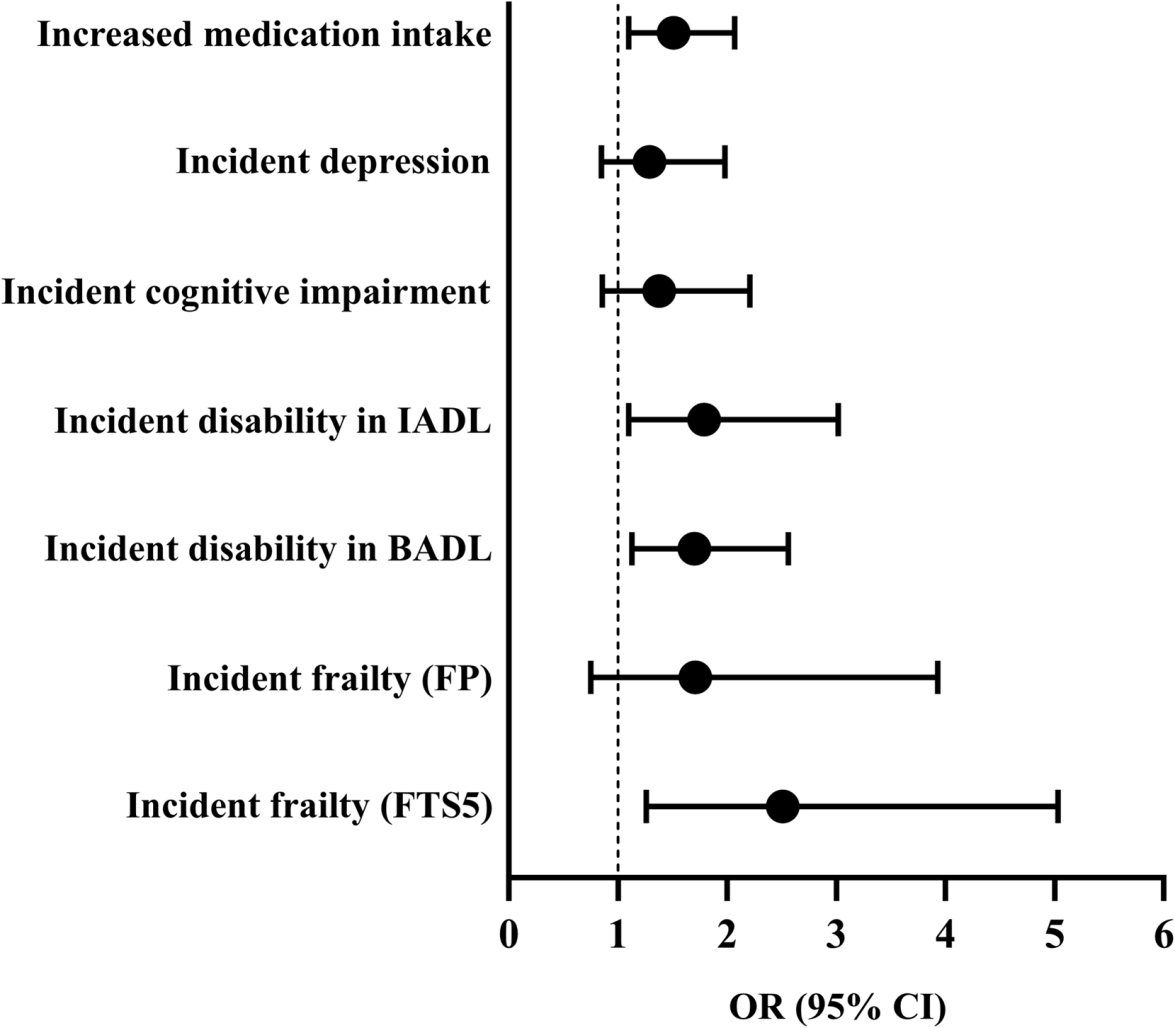
Association of low baseline relative STS power with the development of adverse health outcomes. *Note*: The analysis was adjusted by age, sex, comorbidities and educational level. BADL, basic activities of daily living; CI, confidence interval; FP, frailty phenotype; FTS5, frailty trait scale; IADL, instrumental activities of daily living; OR, odd ratio.

After adjusting for age, sex, Charlson index and educational level, having low relative STS power at baseline was significantly associated with the development of frailty according to the FTS‐5 (OR [95% CI] = 2.51 [1.26–5.03]; *p* = 0.009), disability in BADL (OR [95% CI] = 1.70 [1.13–2.56]; *p* = 0.011), disability in IADL (OR [95% CI] = 1.79 [1.06–3.02]; *p* = 0.030) and medication use (OR [95% CI] = 1.51 [1.10–2.07]; *p* = 0.011) (Figure 4). No associations were found between having low relative STS power at baseline and the development of frailty according to the FP (OR [95% CI] = 1.71 [0.75–3.93]; *p* = 0.202), cognitive impairment (OR [95% CI] = 1.38 [0.86–2.21]; *p* = 0.178) or depression (OR [95% CI] = 1.29 [0.85–1.98]; *p* = 0.236) (Figure 4).

## 4 Discussion

The main findings of this study were that older adults with low relative STS power at baseline showed worse scores in negative health outcomes both at baseline and over the following 5 years. In addition, older adults with low relative STS power at baseline had a higher likelihood of developing adverse health outcomes compared to those without low relative STS power. This study provides novel longitudinal evidence supporting the role of relative STS power as an indicator of future health outcomes in older adults.

This is the first study to demonstrate the long‐term associations of low relative STS power with adverse health outcomes scores both at baseline and after 5 years of follow‐up. Older adults with low relative STS power showed significantly worse values in frailty, BADL and IADL disability, cognitive impairment and medication intake compared to those with normal relative STS power. Notably, these baseline differences in health outcomes between the low and normal‐high relative STS power groups persisted over time. However, no significant differences between older adults with and without relative STS power were observed in depression in either baseline or longitudinal assessments. Furthermore, our findings highlight that older adults with low relative STS power experienced greater deterioration in BADL and IADL disability levels and experienced an increase in medication use, reinforcing the significance of assessing relative STS power and utilizing the previously described cut‐off points over time.

The increase in medication intake and both disabilities (BADL and IADL) in older adults with low relative STS power can be attributed to several factors. Low muscle power has been associated with limitations in essential daily activities, such as walking, stair climbing and rising from a seated position [16, 24, 25]. This reduction of functional capacity and the increased mobility limitations [12] make it more difficult to perform everyday tasks independently, leading to an increase in IADL and BADL disabilities. In addition, low relative STS power has been associated with an increase in the number of comorbidities [12], and it is associated with a higher likelihood of falls [26], which often leads to hospitalizations and injuries, incrementing medication intake.

In this sense, it is important to highlight that this is also the first study that investigates the association of relative STS power with the development of future adverse health outcomes. In this regard, older men and women over the age of 65 with low relative STS power were more likely to develop frailty, disability in BADL and IADL and an increase in medication use compared to their counterparts without low relative STS power. Specifically, older people with low relative STS power had twice the odds of developing frailty compared to those with relative STS power above the established cut‐off points (2.53 W·kg^−1^ for men and 2.01 W·kg^−1^ for women) [16]. Supporting these findings, the cross‐sectional study conducted by Baltasar‐Fernandez et al. [11] reported that older adults with low relative STS power were 5.6 to 6.9 times more likely to be frail. Although our study differs from that of Burbank et al. [27], in key aspects, such as our use of relative rather than absolute STS power, the implementation of sex‐specific cut‐off points and the use of the 30‐s STS test version, our results are in line with their findings, which demonstrated that robust older adults with low relative STS power had a 1.7 times greater probabilities of becoming frail over a 4‐year period, compared to the 2.5 times risk observed in our study over 5 years. One possible explanation involves a multifactorial mechanism. According to Day et al. [28], the oxidation of cysteine residues in crucial proteins of the sarcomere impairs the force and power generation of the sarcomere, which also limits the individuals' mobility [25] and physical functionality [24]. Furthermore, another contributing factor is that individuals with greater disabilities and a sedentary lifestyle tend to have a lower proportion of type II muscle fibres compared to active older adults [29], which compromises their capacity to generate muscle power [30]. Additionally, another studiy has demonstrated that increased fat infiltration is associated with diminished muscle function as well as a higher likelihood of frailty and mortality [31]. This multifactorial decline might increase the likelihood of developing frailty after 5 years.

Moreover, older people with low relative STS power had almost twice the odds of developing disability in BADL and IADL compared to those with normal relative STS power. Similarly, the cross‐sectional study by Bahat et al. [13] demonstrated that low relative STS power was more strongly associated with a higher likelihood of disability in both BADL and IADL than sarcopenia. This finding is consistent with the study by Losa‐Reyna et al. [32], which also observed that low relative STS power was generally more strongly associated with adverse outcomes compared to sarcopenia.

On the other hand, older adults with low relative STS power had a 1.5 times greater likelihood of increasing medication use over the subsequent 5 years. These results were similar to those reported in the cross‐sectional study by Ozkok et al. [33], which showed that individuals with lower STS performance were taking more medications. Finally, despite one cross‐sectional study has highlighted the association between low relative STS power and cognitive function [10], our study did not find a significant association between low relative STS power and the development of cognitive impairment. In this line, a recent cross‐sectional study conducted by our research group showed no association between low relative STS power and depression [16]. This lack of association between low relative STS power and the development of cognitive impairment or depression could be attributed to the absence of sex‐stratified analyses, considering the differences in the development of depressive disorders and cognitive impairment between men and women [34, 35]. Moreover, other factors, such as marital status, anxiety, or genetics, could play a more significant role in the development of depression and cognitive decline than relative STS power itself [35, S5].

The findings of this study highlight the potential clinical utility of assessing relative STS power as an early biomarker of frailty and disability risk. Given its simplicity and feasibility, the STS power test could be incorporated into routine geriatric assessments to identify older adults at higher risk of functional decline. Although STS performance (time or repetitions) and handgrip strength are often used in clinical settings, STS power has shown greater predictive capacity in identifying older adults at increased probabilities of mobility limitations and functional decline [10, 13, 15]. Furthermore, unlike cross‐sectional designs, our findings reinforce the importance of assessing muscle power over time to identify individuals with a higher likelihood of developing disability and frailty. To support its implementation, further research is needed to establish standardized implementation protocols in clinical and community settings to ensure the effective integration of this test into routine geriatric evaluations. Future longitudinal studies should evaluate these parameters in different populations and explore potential interventions to mitigate the decline in muscle power, investigating how these interventions influence adverse health outcomes. Understanding the impact of muscle power and power‐based interventions on health outcomes will be critical for optimizing preventive strategies and promoting healthy aging.

## 5 Study Limitations

This study is not exempt of limitations. First, data loss between the first and second waves of the study, due to mortality, experimental dropout and the exclusion of participants already affected by the syndromes, led to a more homogeneous analysis group. The characteristics of participants who did not complete the follow‐up were significantly worse than those who completed the follow‐up, as they were older, slightly frailer, had greater limitations in ADLs and exhibited higher depression and cognitive impairment. This may introduce some selection bias, potentially limiting the generalizability of our findings to the entire older population. However, this strengthens the robustness of our findings, as significant associations were still observed despite excluding participants with worse health conditions. Additionally, since participants were recruited from the Toledo province (Spain), the generalizability of our findings to older adults in other European or global regions may be limited. Studies conducted in different demographic contexts, such as Latin America, have reported different muscle power values, highlighting the potential influence of regional differences [36]. Finally, although specialized devices such as force plates and inertial sensors are recommended for evaluating muscle power, the use of the STS test in combination with the application of Alcazar's equations has been shown to be a valid and feasible method for this assessment [10], providing an accessible and reliable alternative for evaluating and monitoring muscle power in the clinical setting.

## 6 Conclusion

Our study underscores the significance of assessing relative STS power as an early biomarker of unhealthy aging in older adults. Importantly, it demonstrates that evaluating relative STS power today can help to evaluate the likelihood of developing an adverse health outcome within the next 5 years. This finding is particularly relevant, as there may be older adults with low relative STS power who have not yet manifested any adverse health outcomes and who still have the opportunity to prevent them through appropriate timely interventions. The implementation of decision‐making algorithms [37], alongside with exercise programs aimed at enhancing muscle power [38], can be crucial for restoring relative STS power levels, improving physical function and mitigating frailty, disability and medication intake not only in the short but in the long‐term [39]. Therefore, these strategies could play a vital role in preventing and reducing adverse health outcomes in this vulnerable population, ultimately promoting healthier aging.

## Conflicts of Interest

The authors declare no conflict of interest and certify that they comply with the ethical guidelines for authorship and publishing in the Journal of Cachexia, Sarcopenia, and Muscle [40].

## Supporting information


**Table S1** Baseline characteristics of the participants who completed and did not complete the follow‐up.
**Table S2.** Summary of the linear mixed model analysis.
**Table S3.** Baseline and longitudinal differences in adverse health outcomes scores between older adults with low relative STS power at baseline and those with normal relative STS power at baseline.
**Table S4.** Comparison of longitudinal changes (pre to post) between the low relative STS power group and the normal relative STS power group.
**Table S5.** Unadjusted association of low baseline relative STS power with the development of adverse health outcomes.

**Data S1** Supplementary Information.
