# Supplementary material for: Low Relative Sit‐to‐Stand Power Is Associated With the Development of Adverse Health Outcomes: A 5‐Year Longitudinal Study
Source: J Cachexia Sarcopenia Muscle. 2025 Jun 16;16(3):e13852. doi: 10.1002/jcsm.13852 (PMC12169191; doi:10.1002/jcsm.13852)
Supplement: Supplementary file 1 — Table S1 Baseline characteristics of the participants who completed and did not complete the follow‐up. Table S2. Summary of the linear mixed model analysis. Table S3. Baseline and longitudinal differences in adverse health outcomes scores between older adults with low relative STS power at baseline and those with normal relative STS power at baseline. Table S4. Comparison of longitudinal changes (pre to post) between the low relative STS power group and the normal relative STS power group. Table S5. Unadjusted association of low baseline relative STS power with the development of adverse health outcomes. [file JCSM-16-e13852-s002.docx]

**Supplementary Table S1.** Baseline characteristics of the participants who completed and did not complete the follow-up.

|  | **Completed the follow-up**  **(N = 839)** | | | **Did not complete the follow-up**  **(n = 1037)** | | |
| --- | --- | --- | --- | --- | --- | --- |
|  | **Mean ± SD** | | | **Mean ± SD** | | |
| **Age (years)** | 74.2 | ± | 5.4 | 76.4 | ± | 6.4* |
| **Weight (kg)** | 73.0 | ± | 12.6 | 70.2 | ± | 13.5* |
| **Height (m)** | 1.58 | ± | 0.08 | 1.56 | ± | 0.13* |
| **BMI (kg.m^-2^)** | 29.3 | ± | 4.7 | 29.2 | ± | 4.8 |
| **Relative STS power (W·kg^-1^)** | 2.19 | ± | 0.83 | 2.41 | ± | 0.92 |
| **Low relative STS power (%)** | 54.9 % | | | 59.6 % | | |
| **FTS5 (points)** | 18.9 | ± | 6.8 | 20.4 | ± | 7.9* |
| **Frailty FTS5 (%)** | 21.4 % | | | 28.5%* | | |
| **FP (n of criteria)** | 0.71 | ± | 0.95 | 0.88 | ± | 1.07* |
| **Frailty FP (%)** | 6.0 % | | | 9.5%* | | |
| **Katz index (points)** | 5.85 | ± | 0.66 | 5.62 | ± | 1.10* |
| **Disability in BADL (%)** | 17.2% | | | 22.1%* | | |
| **L&B scale (points)** | 6.38 | ± | 2.0 | 5.77 | ± | 2.29* |
| **Disability in IADL (%)** | 55.1 % | | | 68.5% | | |
| **GDS (points)** | 2.34 | ± | 2.52 | 2.67 | ± | 3.06* |
| **Depression (%)** | 16.1 % | | | 20.2%* | | |
| **MMSE (points)** | 24.2 | ± | 5.01 | 22.5 | ± | 6.05* |
| **Cognitive impairment (%)** | 17.7 % | | | 28.0%* | | |
| **Medications (n)** | 4.7 | ± | 2.6 | 4.7 | ± | 2.7 |
| FTS5: Frailty Trait Scale Short Form; FP: Frailty Phenotype; BADL: basic activities of daily living; L&B scale: Lawton and Brody Scale; IADL: instrumental activities of daily living; GDS: Geriatric Depression Scale; MMSE: Mini-Mental Stated Examination; STS: sit-to-stand; *denotes significant differences compared to those who completed the follow-up (*p*<0.05). | | | | | | |

**Supplementary Table S2.** Summary of the linear mixed model analysis.

|  | **Est.** | **SE** | **95 % CI** | ***p* value** |
| --- | --- | --- | --- | --- |
| **Frailty FTS5 (score)** |  |  |  |  |
| Intersection | 20.379 | 0.405 | (19.58, 21.17) | <0.001 |
| Power group | -4.761 | 0.569 | (-5.88, -3.65) | <0.001 |
| Time | 1.926 | 0.545 | (0.86, 3.00) | <0.001 |
| Power group x time | -1.16 | 0.770 | (-2.67, 0.35) | 0.132 |
| **Frailty FP (n of criteria)** |  |  |  |  |
| Intersection | 0.918 | 0.055 | (0.81, 1.03) | <0.001 |
| Power group | -0.388 | 0.079 | (-0.54, -0.23) | <0.001 |
| Time | 0.06 | 0.074 | (-0.09, 0.21) | 0.418 |
| Power group x time | -0.173 | 0.106 | (-0.38, 0.04) | 0.104 |
| **Katz index (points)** |  |  |  |  |
| Intersection | 5.097 | 0.051 | (5.00, 5.20) | <0.001 |
| Power group | 0.597 | 0.075 | (0.45, 0.75) | <0.001 |
| Time | 0.652 | 0.073 | (0.51, 0.80) | <0.001 |
| Power group x time | -0.366 | 0.107 | (-0.58, -0.16) | 0.001 |
| **L&B scale (points)** |  |  |  |  |
| Intersection | 5.345 | 0.111 | (5.13, 5.56) | <0.001 |
| Power group | 1.314 | 0.163 | (0.99, 1.63) | <0.001 |
| Time | 0.724 | 0.158 | (0.41, 1.03) | <0.001 |
| Power group x time | -0.591 | 0.231 | (-1.05, -0.14) | 0.011 |
| **MMSE (points)** |  |  |  |  |
| Intersection | 21.056 | 0.278 | (20.51, 21.60) | <0.001 |
| Power group | 2.389 | 0.408 | (1.59, 3.19) | <0.001 |
| Time | 2.681 | 0.406 | (1.89, 3.48) | <0.001 |
| Power group x time | -1.133 | 0.593 | (-2.30, 0.03) | 0.056 |
| **GDS (points)** |  |  |  |  |
| Intersection | 4.182 | 0.127 | (3.93, 4.43) | <0.001 |
| Power group | -0.59 | 0.185 | (-0.95, -0.23) | 0.001 |
| Time | -1.584 | 0.181 | (-1.94, -1.23) | <0.001 |
| Power group x time | -0.037 | 0.265 | (-0.56, 0.48) | 0.888 |
| **Medication intake (n)** |  |  |  |  |
| Intersection | 6.498 | 0.142 | (6.22, 6.78) | <0.001 |
| Power group | -1.398 | 0.208 | (-1.81, -0.99) | <0.001 |
| Time | -1.503 | 0.202 | (-1.90, -1.11) | <0.001 |
| Power group x time | 0.55 | 0.299 | (-0.04, 1.14) | 0.066 |
| Est: estimates; SE: standard error; FTS5: Frailty Trait Scale Short Form; FP: Frailty Phenotype; BADL: basic activities of daily living; L&B scale: Lawton and Brody Scale; IADL: instrumental activities of daily living; GDS: Geriatric Depression Scale; MMSE: Mini-Mental Stated Examination.  ***Note:*** Power group: 1 = Low relative STS power, 0 = Normal relative STS power. Time: 1 = baseline assessment, 2 = follow-up assessment (after 5 years).  The analysis was adjusted for age, sex, comorbidities, educational level and baseline values. | | | | |

**Supplementary Table S3.** Baseline and longitudinal differences in adverse health outcomes scores between older adults with low relative STS power at baseline and those with normal relative STS power at baseline.

|  | | **Low relative STS power** | | | **Normal relative STS power** | | **Low *vs.* Normal relative STS power** | | |
| --- | --- | --- | --- | --- | --- | --- | --- | --- | --- |
|  |  | **Mean** | | **SE** | **Mean** | **SE** | **∆** | **95% CI** | ***p*** |
| **FTS5 (score)** | |  | |  |  |  |  |  |  |
|  | Pre | 22.3 | | 0.37 | 16.4 | 0.37 | 5.9 | 4.90 to 6.94 | <0.001 |
|  | Post | 20.4 | | 0.41 | 15.6 | 0.40 | 4.8 | 3.65 to 5.88 | <0.001 |
|  | Pre to post (∆) | -1.93* | | 0.54 | -0.77 | 0.54 |  | | |
| **Frailty phenotype (n of criteria)** | |  | |  |  |  |  |  |  |
|  | Pre | 0.98 | | 0.05 | 0.42 | 0.05 | 0.56 | 0.42 to 0.70 | <0.001 |
|  | Post | 0.92 | | 0.6 | 0.53 | 0.06 | 0.39 | 0.23 to 0.54 | <0.001 |
|  | Pre to post (∆) | 0.06 | | 0.07 | -0.11 | 0.07 |  | | |
| **Katz index (points)** | |  | |  |  |  |  |  |  |
|  | Pre | 5.75 | | 0.05 | 5.98 | 0.06 | - 0.23 | -0.38 to -0.08 | 0.002 |
|  | Post | 5.10 | | 0.05 | 5.69 | 0.06 | - 0.60 | -0.74 to - 0.45 | <0.001 |
|  | Pre to post (∆) | 0.65* | | 0.07 | 0.29* | 0.08 |  |  |  |
| **L&B scale (points)** | |  |  |  |  |  |  |  |  |
|  | Pre | 6.07 | | 0.11 | 6.79 | 0.12 | -0.72 | -1.05 to -0.40 | <0.001 |
|  | Post | 5.35 | | 0.11 | 6.66 | 0.12 | -1.31 | -1.63 to -0.99 | <0.001 |
|  | Pre to post (∆) | 0.72* | | 0.16 | 0.13 | 0.17 |  | | |
| **MMSE (points)** | |  | |  |  |  |  |  |  |
|  | Pre | 23.7 | | 0.30 | 25.0 | 0.31 | -1.26 | -2.10, -0.41 | 0.004 |
|  | Post | 21.1 | | 0.28 | 23.4 | 0.30 | -2.39 | -3.19, -1.59 | <0.001 |
|  | Pre to post (∆) | 2.68 | | 0.41 | 1.55 | 0.43 |  |  |  |
| **GDS (points)** | |  | |  |  |  |  |  |  |
|  | Pre | 2.60 | | 0.13 | 1.97 | 0.14 | 0.63 | 0.25, 1.00 | <0.001 |
|  | Post | 4.18 | | 0.13 | 3.59 | 0.14 | 0.59 | 0.23, 0.95 | <0.001 |
|  | Pre to post (∆) | 1.58* | | 0.18 | 1.62* | 0.19 |  |  |  |
| **Medication intake (n)** | |  | |  |  |  |  |  |  |
|  | Pre | 5.00 | | 0.14 | 4.15 | 0.16 | 0.85 | 0.43, 1.27 | <0.001 |
|  | Post | 6.50 | | 0.14 | 5.10 | 0.15 | 1.40 | 0.99, 1.81 | <0.001 |
|  | Pre to post (∆) | 1.50* | | 0.20 | 0.95* | 0.22 |  |  |  |

PRE: baseline values; POST: follow-up values; SE: standard error; CI: confidence intervals; FTS5: Frailty Trait Scale Short Form; L&B scale: Lawton and Brody Scale; GDS: Geriatric Depression Scale; MMSE: Mini-Mental Stated Examination; STS: sit-to-stand;

*Note:* The analysis was adjusted for age, sex, comorbidities, educational level and baseline values. *denotes significant pre to post changes within the same group (*p*<0.05).

**Supplementary Table S4.** Comparison of longitudinal changes (pre to post) between the low relative STS power group and the normal relative STS power group.

|  | **Low relative STS power ∆ (pre to post) *vs.* Normal relative STS power ∆ (pre to post)** | | | |
| --- | --- | --- | --- | --- |
|  | **∆** | **SE** | **95 % CI** | ***p* value** |
| **Frailty FTS5 (score)** | -0.43 | 0.55 | - 1.51 to 0.65 | 0.438 |
| **Frailty FP (n of criteria)** | -0.04 | 0.09 | -0.22 to 0.13 | 0.631 |
| **Katz index (points)** | -0.38 | 0.09 | -0.55 to -0.21 | <0.001 |
| **L&B scale (points)** | -0.64 | 0.17 | -0.98 to -0.30 | <0.001 |
| **MMSE (points)** | -0.93 | 0.41 | -1.74 to -0.12 | 0.024 |
| **GDS (points)** | 0.04 | 0.24 | -0.42 to 0.51 | 0.855 |
| **Medication intake (n)** | 0.47 | 0.21 | 0.07 to 0.88 | 0.022 |

SE: standard error; CI: confidence interval; FTS5: Frailty Trait Scale Short Form; L&B scale: Lawton and Brody Scale; GDS: Geriatric Depression Scale; MMSE: Mini-Mental Stated Examination; STS: sit-to-stand.

*Note:* The analysis was adjusted for age, sex, comorbidities, educational level.

**Supplementary Table S5.** Unadjusted association of low baseline relative STS power with the development of adverse health outcomes.

|  | **Unadjusted model** | | | |
| --- | --- | --- | --- | --- |
|  | **OR** | **95 % CI** | ***p* value** | |
| **Frailty FTS5** | 2.29 | 1.18 – 4.47 | 0.015 | |
| **Frailty FP** | 2.06 | 0.93 – 4.59 | 0.077 | |
| **Cognitive Impairment** | 1.45 | 0.94 – 2.25 | 0.095 | |
| **Depression** | 1.31 | 0.87 – 1.97 | 0.200 | |
| **Disability in BADL** | 2.02 | 1.40 – 2.92 | <0.001 | |
| **Disability in IADL** | 2.33 | 1.43 – 3.78 | <0.001 | |
| **Medication use** | 1.48 | 1.08 – 2.03 | 0.016 | |
| OR: odd ratio; CI: confidence interval; FTS5: Frailty Trait Scale; FP: Frailty Phenotype; BADL: basic activities of daily living; IADL: instrumental activities of daily living. | | | |  |
