# Supplementary material for: Low Relative Sit‐to‐Stand Power Is Associated With the Development of Adverse Health Outcomes: A 5‐Year Longitudinal Study
Source: J Cachexia Sarcopenia Muscle. 2025 Jun 16;16(3):e13852. doi: 10.1002/jcsm.13852 (PMC12169191; doi:10.1002/jcsm.13852)
Supplement: Supplementary file 2 — Data S1 Supplementary Information. [file JCSM-16-e13852-s001.docx]

**Supplementary references**

S1. Khan SS, Singer BD, Vaughan DE. Molecular and physiological manifestations and measurement of aging in humans. Aging Cell. 2017;16:624-33.

S2. Husted KLS, Brink-Kjær A, Fogelstrøm M, Hulst P, Bleibach A, Henneberg K, et al. A Model for Estimating Biological Age From Physiological Biomarkers of Healthy Aging: Cross-sectional Study. JMIR Aging. 2022;5:e35696.

S3. Charlson ME, Pompei P, Ales KL, MacKenzie CR. A new method of classifying prognostic comorbidity in longitudinal studies: development and validation. J Chronic Dis. 1987;40:373-83.

S4. Meulemans L, Alcazar J, Alegre LM, Dalle S, Koppo K, Seghers J, et al. Sensor- and equation-based sit-to-stand power: The effect of age and functional limitations. Exp Gerontol. 2023;179:112255.

S5. Hung YC, Chen YH, Lee MC, Yeh CJ. Effect of Spousal Loss on Depression in Older Adults: Impacts of Time Passing, Living Arrangement, and Spouse's Health Status before Death. Int J Environ Res Public Health. 2021;18:
